# Dynamics of microbial populations mediating biogeochemical cycling in a freshwater lake

**DOI:** 10.1186/s40168-018-0556-7

**Published:** 2018-09-18

**Authors:** Keith Arora-Williams, Scott W. Olesen, Benjamin P. Scandella, Kyle Delwiche, Sarah J. Spencer, Elise M. Myers, Sonali Abraham, Alyssa Sooklal, Sarah P. Preheim

**Affiliations:** 1 Department of Environmental Health and Engineering, Johns Hopkins University, Baltimore, MD USA; 2 Department of Biological Engineering, Massachusetts Institute of Technology, Cambridge, MA USA; 3 Department of Civil and Environmental Engineering, Massachusetts Institute of Technology, Cambridge, MA USA; 4 Present address: Department of Immunology and Infectious Diseases, Harvard T. H. Chan School of Public Health, Boston, MA USA; 5 Present address: Institute of the Environment and Sustainability, University of California, Los Angeles, CA USA; 6 Present address: Earth and Environmental Sciences, Columbia University, Palisades, NY USA; 7 Present address: Oregon Water Resources Department, Salem, OR USA; 8 Harvard John A. Paulson School of Engineering and Applied Sciences, Cambridge, MA USA

**Keywords:** Biogeochemical model, 16S rRNA gene sequencing, Metagenome-assembled genome

## Abstract

**Background:**

Microbial processes are intricately linked to the depletion of oxygen in in-land and coastal water bodies, with devastating economic and ecological consequences. Microorganisms deplete oxygen during biomass decomposition, degrading the habitat of many economically important aquatic animals. Microbes then turn to alternative electron acceptors, which alter nutrient cycling and generate potent greenhouse gases. As oxygen depletion is expected to worsen with altered land use and climate change, understanding how chemical and microbial dynamics impact dead zones will aid modeling efforts to guide remediation strategies. More work is needed to understand the complex interplay between microbial genes, populations, and biogeochemistry during oxygen depletion.

**Results:**

Here, we used 16S rRNA gene surveys, shotgun metagenomic sequencing, and a previously developed biogeochemical model to identify genes and microbial populations implicated in major biogeochemical transformations in a model lake ecosystem. Shotgun metagenomic sequencing was done for one time point in Aug., 2013, and 16S rRNA gene sequencing was done for a 5-month time series (Mar.–Aug., 2013) to capture the spatiotemporal dynamics of genes and microorganisms mediating the modeled processes. Metagenomic binning analysis resulted in many metagenome-assembled genomes (MAGs) that are implicated in the modeled processes through gene content similarity to cultured organism and the presence of key genes involved in these pathways. The MAGs suggested some populations are capable of methane and sulfide oxidation coupled to nitrate reduction. Using the model, we observe that modulating these processes has a substantial impact on overall lake biogeochemistry. Additionally, 16S rRNA gene sequences from the metagenomic and amplicon libraries were linked to processes through the MAGs. We compared the dynamics of microbial populations in the water column to the model predictions. Many microbial populations involved in primary carbon oxidation had dynamics similar to the model, while those associated with secondary oxidation processes deviated substantially.

**Conclusions:**

This work demonstrates that the unique capabilities of resident microbial populations will substantially impact the concentration and speciation of chemicals in the water column, unless other microbial processes adjust to compensate for these differences. It further highlights the importance of the biological aspects of biogeochemical processes, such as fluctuations in microbial population dynamics. Integrating gene and population dynamics into biogeochemical models has the potential to improve predictions of the community response under altered scenarios to guide remediation efforts.

**Electronic supplementary material:**

The online version of this article (10.1186/s40168-018-0556-7) contains supplementary material, which is available to authorized users.

## Background

Biogeochemical processes in hypoxic and anoxic environments can feedback on changes to local and global ecosystems, so understanding how they are regulated will serve to aid remediation efforts, model climate change, and protect public health. Oxygen depletion is a major impairment to water bodies in the USA [1] and is likely to become more severe in a warming climate [2]. Oxygen depletion deprives economically important aquatic organisms of vital habitat. Often remediation efforts designed to improve the ecosystem are not sufficient to restore esthetics, biodiversity, and functionality of impacted water bodies [3]. Oxidation-reduction processes initiated upon oxygen depletion can influence the mobility of toxic substances within the water body, such as uranium [4] and arsenic [5]. Migration of these substances into surface or groundwater can have adverse effects on human health. Low-oxygen concentrations can also alter the availability of key nutrients, nitrogen and phosphorous [6–9], that can impact the productivity of the ecosystem. Finally, microbial processes such as methanogenesis, methane oxidation, and denitrification mediate the production and consumption of methane and nitrous oxide that could impact atmospheric levels [10]. Improving predictive models of microbial processes in low-oxygen environments will provide a way to test the community response to altered environmental conditions.

Population dynamics are not typically used to improve predictive models of microbial processes because of the difficulty of determining in situ relationships between populations and processes. Microbial processes, especially in anoxic environments, are traditionally thought of as a “black-box,” where the identity of the microorganisms involved is ignored [e.g., [11]]. The high degree of functional redundancy was thought to make identity irrelevant [12]. A number of studies of host-associated microbiomes (algae [13], sponges [14], and human [15]) note large changes in taxonomic composition associated with relative invariance in functional gene content. This suggests that changes in community membership do not yield meaningful differences in community function. Functional biomarker genes have been used to drive biogeochemical models of an oxygen minimum zone [16] and a eutrophic estuary [17]. An underlying assumption in such efforts is that the rest of the genomic repertoire of mostly uncultivated populations is irrelevant for understanding their effect on biogeochemical process.

However, there is consensus in macroecology that species’ functional characteristics significantly alter ecosystem energy and material flow via positive and negative interactions with other species [18]. Examples of such events include the introduction of the actinorhizal nitrogen fixer *Myrica faya* in Hawaii [19] and the reestablishment of wolf populations in Yellowstone [20]. In these examples, the spatiotemporal dynamics of invasive species or keystone predators drive change to a greater extent than abiotic factors.

In microbial ecology of low-oxygen environments, less is known about how the unique characteristics of microbial populations influence energy and material flow. Yet, examples suggest microbial population functional properties can be important. Phytoplankton functional types (e.g., diatoms, cyanobacteria, chlorophytes, and coccolithophores) impact carbon cycling differently enough to warrant individual parameters in many biogeochemical models [21]. Competition for electron donors between *Rhodoferax* and *Geobacter* may influence uranium bioremediation because *Geobacter* can reduce uranium, while *Rhodoferax* cannot [22]. Since syntrophic and competitive interactions are common between microbial populations in anoxic environments [23, 24], both interaction and successional dynamics could have a strong impact on overall biogeochemistry. More work is needed to identify these unique population characteristics and understand how they affect predictive models.

The first hurdle in understanding the impact of population-specific traits and interactions on ecosystems is identifying them in natural environments. This often requires inference rather than direction observation. Directly observing the functional capabilities and interactions of microbial populations in the environment provides the most accurate way to probe interactions [25], especially when organisms are physically associated [24] and metabolites can be traced with the use of isotopic signatures (e.g., [26, 27]). However, direct evidence requires substantial prior knowledge, can be limited to certain types of interactions and often requires experimental manipulation. Thus, functional capabilities and ecological interactions are often inferred through indirect evidence. Partial genome reconstruction facilitated by metagenomic binning has vastly improved our understanding of the genetic potential of uncultured microorganisms (e.g., [28, 29]). Competition and syntrophic interactions can also be inferred through co-occurrence patterns [30] and time-series analysis [31]. Bioinformatic inference allows for the investigation of a less well-characterized functions and interactions in ecosystems.

The Upper Mystic Lake’s unique chemistry, simple hydrology, and previously characterized microbial community make it amenable to investigations of microbial gene and population dynamics. The chemical environment of the Upper Mystic Lake is impacted by pollution from the Industri-Plex and Wells G & H Superfund sites (Woburn, MA). Contaminants from the Superfund site, such as nitrogen, iron, and arsenic, have complex interconnections in the lake. Specifically, nitrate controls arsenic [32] and phosphate [33] mobilization through microbially mediated iron oxidation with nitrate. Methane concentrations are also elevated in the lake, resulting in methane release to the atmosphere through bubbling that is correlated with changes in hydrostatic pressure [34]. Microorganisms mediating oxidation-reduction reactions of nitrogen, iron, and methane were previously characterized using a 16S rRNA gene survey and a biogeochemical model [35]. The microorganisms mediating biogeochemical processes in the lake co-occur with other microbes not directly mediating the modeled processes, suggesting syntrophic interactions play an important part in these processes as well.

Here, we compare gene and population spatiotemporal dynamics to a biogeochemical model in Upper Mystic Lake. We use 16S rRNA gene sequencing and metagenomic binning to infer capabilities. Population dynamics and implications of potential interactions are compared with a computational model capturing the ecosystem-level biogeochemistry of the lake. This framework allows us to identify capabilities and interactions that could substantially alter the predicted chemistry of the lake and observe the dynamics of dominant populations related to predicted biogeochemical cycling. Elucidating the gene organization within and between populations could improve our ecosystem-level understanding of biogeochemical processes and the factors that control them.

## Results

### Biogeochemical process predictions from a previous model largely explain gene distribution

To better understand the relationship between biogeochemical processes and the distribution of genes within the lake, we compared the distributions of key genes to the rates of the corresponding processes predicted by a biogeochemical model. The biogeochemical model was developed from a reactive transport model coupling major chemical cycles [11] and adapted for this lake ecosystem [35]. We assume that dispersion below the thermocline distribute cells evenly throughout water column. We assume that the presence of diagnostic genes mediating biogeochemical processes in the model allow cells to actively reproduce in proportion to energy availability and all other physical or ecological processes (e.g., predation, mortality) impact microorganisms evenly throughout the water column. This would result in a relative gene distribution proportional to the relative rates of the biogeochemical processes they mediate. While other recent biogeochemical models use gene abundances to drive biogeochemical rates [16, 17], our model was calibrated independent from observed gene abundances. Previously, the model predictions explained the distribution of populations inferred to have specific capabilities through phylogenetic similarity to cultured populations or through a novel single-cell method to fuse functional and phylogenetically informative genes, Emulsion, Paired Isolation, and Concatenation PCR (epicPCR; [36]). In this analysis, we compare model predictions to distribution of genes in shotgun metagenomic data involved in methane, sulfur, nitrogen, and iron cycling in the lake.

**Fig. 1:**
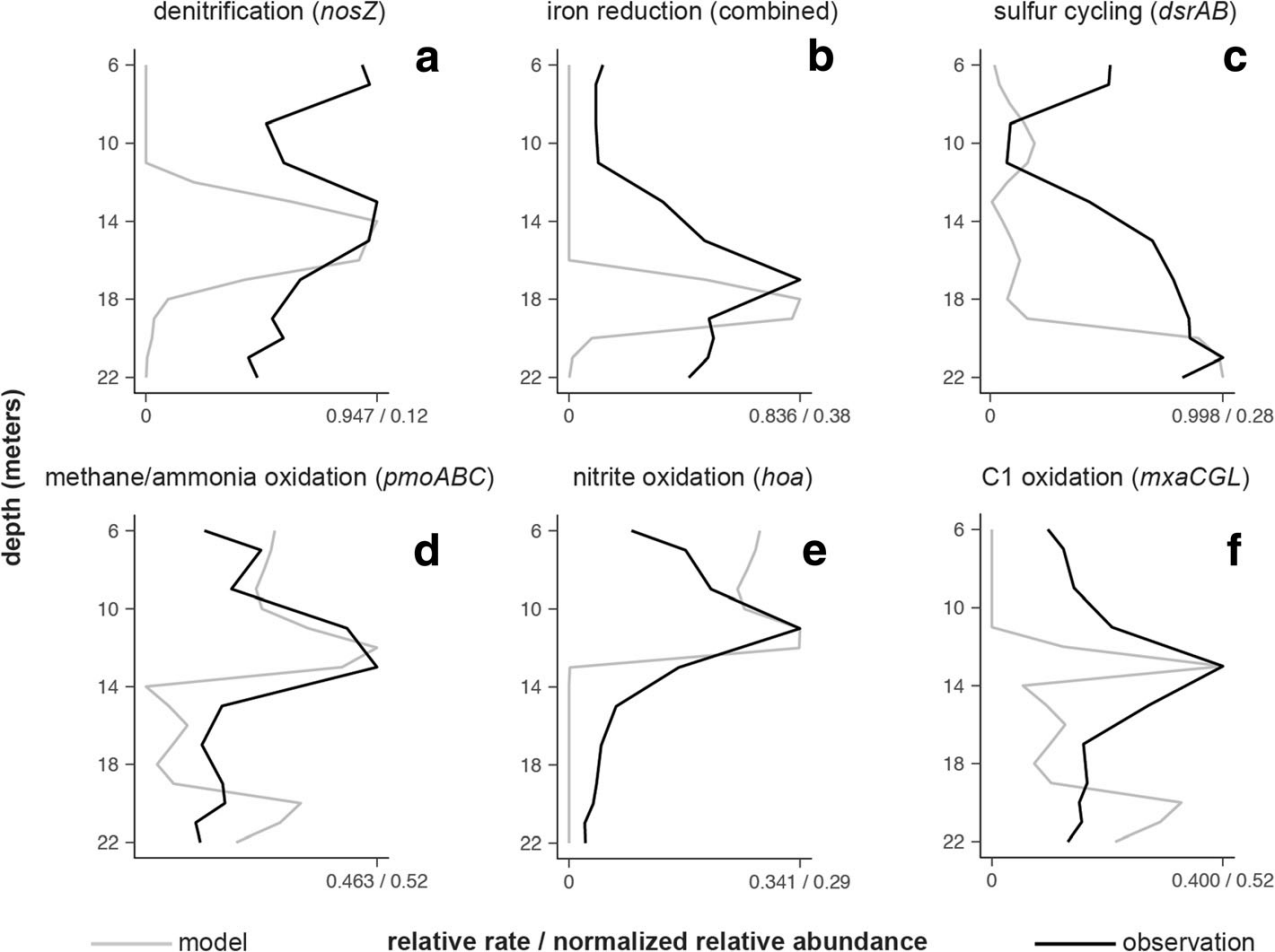
Distribution of genes (black lines, normalized relative abundance) and their correspondence with modeled processes (gray lines, relative rate) suggest that the model captures the major factors influencing the distribution of most genes in the lake, except genes involved in sulfur cycling. Modeled rates are identical to those published in a previous analysis, which were not calibrated to match gene distributions. Observations represent the following genes and corresponding processes: **a** *nosZ* genes with associated modeled processes heterotrophic and autotrophic denitrification, combined; **b** genes involved in iron reduction in *Geobacter* and *Rhodoferax* and modeled heterotrophic iron reduction; **c** *dsrB* genes and modeled heterotrophic sulfate reduction and autotrophic sulfide oxidation, combined; **d** *pmoABC* genes and modeled methane oxidation and nitrification, combined; **e** *hoa* genes and modeled nitrification; and **f** *mxaG* genes and modeled methane oxidation (using both oxygen and sulfate)

While the model predictions align with most gene observations from metagenomic data, the model predictions do not explain the overall distribution of key genes involved in sulfur cycling, and to a lesser extent methane cycling, in the lake (Fig. 1c, f). The model captured the major trends in denitrification (*nosDZ*), iron reduction (*Geobacter* and *Rhodoferax* iron-reducing genes), and nitrification (*hoa*), through the distribution of diagnostic genes for each process. However, the model did very poorly in capturing the overall distribution of the diagnostic gene for dissimilatory sulfate reduction and sulfur oxidation (*dsrAB*). Additionally, the overall trends in the diagnostic genes involved in both methane and ammonia oxidation (*pmoABC*) and genes involved in methane oxidation alone (*mxaK*) do not display a peak near the bottom which is mirrored by the model (Fig. 1f). These results suggest that methane and sulfur cycling are not properly explained in the model or that our experimental gene distributions are determined by other factors not related to energy availability, including organisms that harbor metabolic genes that are not actively used in energy generation (i.e., metabolic versatility).

### Metagenome-assembled genomes provide insight into population capabilities

The coupling of genes within genomes of dominant microorganisms in the lake can provide insight into the factors that influence gene distribution. It can provide an estimate of the metabolic capabilities of abundant microorganisms and identify metabolic versatility and redox processes that were not previously included in the model. We used metagenomic binning to partially reconstruct genomes of lake microorganisms from Aug. 2013. Metagenomic assembly and binning resulted in 87 metagenomic bins, or metagenome-assembled genomes (MAGs) with completeness greater than 70% (median 84.4%) and less than 10% contamination (Additional file 1). The metabolic capabilities associated with MAGs were further characterized by clustering them to each other and to genomes in the Kyoto Encyclopedia of Genes and Genomes (KEGG) database (Additional files 2 and 3) according to shared gene content. Finally, the presence of key genes and pathways in energy metabolism was used to confirm the metabolic capabilities associated with each MAG (Additional file 4).

Insight into the spatiotemporal dynamics of populations [i.e., operational taxonomic units (OTUs) from distribution-based clustering] over the course of the entire season (March–Aug., 2013) was assessed by amplicon libraries of the 16S rRNA gene. To gain insight into how metabolic capabilities shape population dynamics in relation to the model predictions, we matched OTUs to MAGs to provide confirmation about the role of specific OTUs in the processes described in the model. During the normal course of de-novo assembly and the binning, highly conserved genes with uniquely divergent tetranucleotide frequencies, such as 16S rRNA genes [37], are prone to being misassembled or misplaced during binning. Thus, an additional algorithm was needed to integrate the functional repertoire of MAGs to the extended spatiotemporal dynamics of OTUs. We used Euclidean distances between normalized abundance vectors and the shared fraction of the taxonomic classification hierarchy to connect OTUs to MAGs. The unification of data types facilitated comparisons between the changing abundances of populations in time and space and related modeled processes over the same scope.

We employed many quality control measures to ensure our method for matching OTUs to MAGs was robust. First, the few 16S rRNA gene sequences found within the MAGs were used to validate algorithm parameters. Additionally, we confirmed that bins containing the genome of positive control samples (*Escherichia coli*, *Microcystis aeruginosa*) had the correct 16S rRNA gene sequences. We also used 16S rRNA gene sequences experimentally determined to be associated with *dsrB* through epicPCR [36] as a validation (i.e., seq106 should have *dsrB*, seq301 should not have *dsrB*). Finally, as an additional quality control, we required that the 16S rRNA gene sequence matching the MAG was identical in both an amplicon dataset generated independently of the metagenomic libraries and from an analysis of 16S rRNA gene sequences obtained from the metagenomic libraries directly. This minimizes any bias associated with the different library preparation approaches. Not all MAGs were assigned specific OTU sequences with these quality control measures, but a number of OTUs were assigned to MAGs capable of mediating many of the modeled processes (Table 1).

**Table 1:** Populations (OTUs) and associated genomes (MAGs) implicated in mediating processes in the biogeochemical model

Biogeochemical process	Classification	OTU/MAG	Metabolic versatility (genes)
Iron oxidation	*Sulfuricella denitrificans/Sideroxydans lithotrophicus* ES-1	seq6/bin.59	Sulfur cycling (*dsrAB*); Denitrification (*nosZ*)
Sulfur oxidation	*Sulfuritalea hydrogenivorans*	seq335/bin.15	Denitrification (*nosZ*)
Methane oxidation	*Methylobacter tundripaludum*	seq172/bin.78	Denitrification (*nxrAB, nirK, norBC*)
Methanol oxidation	*Methyloterna versitalis*	seq3/bin.71	NA
Ammonia oxidation	*Nitrosospira briensis*	seq39/bin.19	Denitrification (*nirK*)
Denitrification	*Bacteroidetes*	Various	NA
Iron reduction	*Rhodoferax ferrireducens* T118	seq12/bin.4	Denitrification (*nxrAB, nirS*)
Iron reduction	*Geobacter*	seq228_1/bin.34	NA
Sulfate reduction	*Desulfatirhabdium butyrativorans* strain HB1	seq106/bin.25	NA

### Population capabilities inferred from partial genome reconstructions with metagenomic binning

**Fig. 2:**
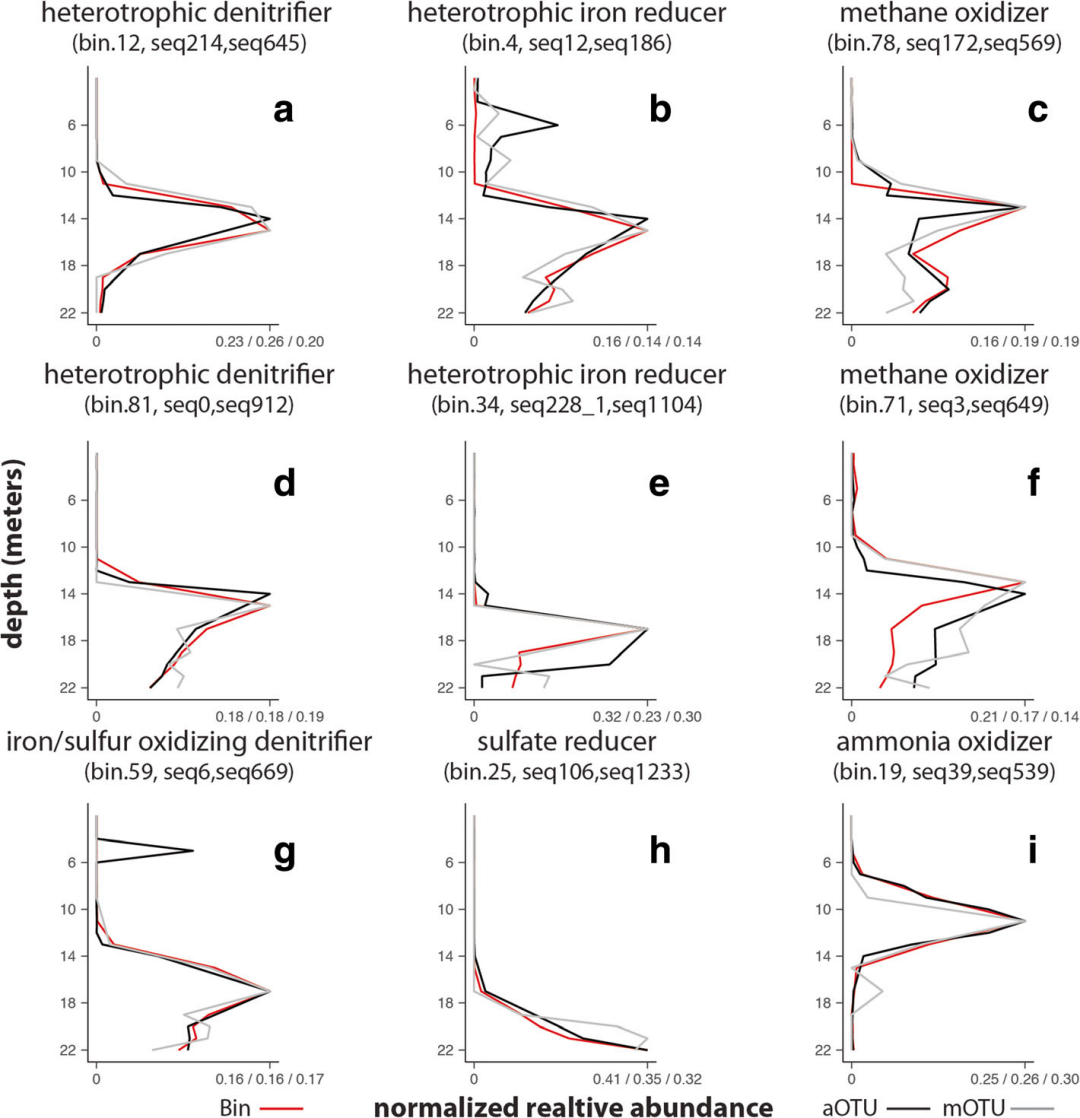
**a**–**i** Distribution MAGs (bins) and matched OTUs within the water column. To match OTUs with MAGs, the MAG distribution (red) had to align with both the amplicon OTU (aOTU; black) and metagenomic OTU (mOTU; gray) distributions of the same sequence. From the most abundant OTUs, these OTUs matched the MAGs with a similar distribution and classification. The MAG characteristics, including similarity to cultured microorganisms with the same characteristic and presence of the genes in the MAG, support the role of these OTUs in the modeled process in the lake

We identified multiple OTUs that matched MAGs associated with the modeled processes for denitrification, iron reduction, sulfate reduction, methanotrophy, nitrification, and iron and sulfur oxidation (Fig. 2). Five MAGs matched key populations identified previously, supporting their putative role in biogeochemical cycling. One MAG (bin.19) containing genes for ammonia oxidation clustered with other autotrophic ammonia-oxidizing KEGG genomes and matched the key population previously identified as an ammonia oxidizer (seq39). This bin also contained nitrite reductase (*nirK*), suggesting the capability of nitrifier denitrification [38, 39]. A MAG (bin.25) containing *dsrAB* genes and clustering with other sulfate reducing organisms matched the sequence experimentally identified as a sulfate reducer (seq106). One MAG (bin.4) containing genes involved in iron reduction in *Rhodoferax* and clustering with KEGG genome *Rhodoferax ferrireducens* matched an OTU similar to *Rhodoferax ferrireducens* (seq12). In addition to genes involved in iron reduction, the *Rhodoferax* MAG possessed genes involved in denitrification (*nxrAB*, *nirS*). A different iron-reducing MAG (bin.34) closely linked to *Geobacter* spp*.* initially did not appear to match well with any OTUs. In this case, the short (102 bp) amplicon sequences used to match to the metagenomic libraries’ read sequences was not long enough to resolve the diversity of *Geobacter* populations well in these samples. Extending the amplicon analysis to 250 bases revealed two distinct sequences with unique distributions represented by one OTU (seq228_1; Additional file 5), one of which could be assigned to bin.34 with a Euclidean distance within the cutoff (0.04). Finally, an OTU matching the key population for iron oxidation with nitrate similar to *Sideroxydans lithotrophicus* ES-1 (seq6) matched a MAG (bin.59) clustering with both iron and sulfur-oxidizing KEGG genomes *Sulfuricella denitrificans* skB26 and *Sideroxydans lithotrophicus* ES-1. Because genes involved in iron oxidation are not well characterized, it is difficult to determine whether these MAGs contain the necessary genes for iron oxidation. *Sideroxydans lithotrophicus* ES-1 is known to contain *dsrAB* genes involved in sulfur oxidation [40], adding to metabolic flexibility. These observations largely support our previous analysis that these populations are involved in key biogeochemical transformations in the lake.

Other OTU sequences matching MAGs with genes and genomic characteristics supportive of their role in the modeled processes were also found in the lake. Four MAGs (bin.46, bin.48, bin.81, bin.12/bin.60) with matching *Bacteroidetes* OTUs (seq280, seq433, seq0, seq214, resp.) had at least one gene involved in denitrification (i.e., *nosZ*) and clustered near other heterotrophic KEGG genomes (Additional file 2). None of these or any other assembled MAGs had a complete denitrification pathway. The two other MAGs associated with denitrification coupled to sulfur oxidation also match two OTUs classified as *Betaproteobacteria* (bin.15->seq335; bin.73->seq1088). A MAG implicated in methanotrophy (bin.78) aligns with OTUs similar to a methanotroph *Methylobacter tundripaludum* (seq172). Another MAG (bin.71), clustering near methylotrophs genomes, contained genes involved in methanol metabolism and carbon fixation and matched an OTU (seq3) similar to *Methyloterna versitalis*.

All bins passing our quality control metrics were classified as *Bacteria*. The dominance of bacterial bins in the final dataset agrees with overall classification from the shotgun metagenomic data. Nearly 63.8% of assembled contigs could not be matched to known sequences or were matched to uninformative sequences. The rest were classified as *Bacteria* (96.8%), *Eukaryota* (2.8%), *Archaea* (0.1%), or viral (0.3%). Some preliminary bins, which failed our quality control measures, contained non-bacterial sequences. A single preliminary bin, annotated only at the level of kingdom as *Archaea*, fell just short of the completeness criterion. Upon further inspection of this bin, none of the functional genes used in this study were detected in the bin. Five preliminary bins were annotated as primarily eukaryotic, but the phyla assigned to individual contigs within those bins varied widely. Another small preliminary bin contained three 40–50 kbp viral contigs, two of which were classified as *Podoviridae*. All three peaked in abundance between 9 m and 13 m and were twice as abundant as the most abundant bacterial MAGs.

### Identification of potentially important pathways missing from the model

From the metagenomic bins, we were able to identify two metabolic processes that were not previously included in the model. A number of MAGs (bin.59, bin.15, bin.73) clustered to the KEGG genomes of freshwater sulfur-oxidizing autotrophs capable of denitrification, *Sulfuritalea hydrogenivorans* [41], and *Sulfuricella denitrificans* [42]. These MAGs contained the diagnostic genes for carbon fixation (*rbcLS*), sulfur cycling (*dsrAB*), and denitrification (*nosZ*). One MAG (bin.59) also clustered with iron oxidizing autotroph *Sideroxydans lithotrophicus* ES-1. Bin.59 is the most relatively abundant bin from 17 to 21 m depth. Thus, if this MAG is associated with iron oxidation, it also contains sulfur-cycling genes that add to metabolic flexibility, which was previously observed [40]. The model did not include sulfide oxidation with nitrate, so it is unclear from the current model predictions where this process is expected to occur within the water column to compare to the MAG distributions.

Another metagenomic bin provides evidence for coupling methane oxidation to nitrate and/or nitrite reduction, which was not included in the model. A number of MAGs cluster near KEGG genome *Methylomicrobium alcaliphilum* [T01649; [43]]. One of these MAGs (bin.78) contains methane-oxidizing genes (*pmoA)* along with genes involved in nitrate (*nxr*), nitrite (*nir*), and nitric oxide (*nor*) reduction. Coupling methane oxidation with nitrate reduction under hypoxic conditions has been demonstrated in other *Methylomicrobium* populations [44]. The model does not include this process, so we cannot compare where this process would be expected, given the chemistry.

Although this analysis does not confirm that sulfur or methane oxidation is coupled to denitrification within this system, the model can be used to determine whether these processes are favorable given the chemical environment, and if so, what the impact would be to overall biogeochemistry. Sulfur and methane oxidation with nitrate were the two processes not included in the original reactive transport model [11]. However, both processes have since been shown to be widespread and important [45–47].

### Competing denitrification processes could substantially alter lake biogeochemistry

To determine the potential impact of sulfur and methane oxidation coupled to denitrification in the lake, the model was updated and calibrated to match the chemical and gene observations. We calibrated model parameters to match both chemistry and key genes involved in each processes, an approach used previously [16]. Using previously published values, the model fit with a mean absolute error (MAE) of 0.205 to the concentrations of sulfate, dissolved oxygen, nitrate, ferrous iron, and gene abundance across the 3-month time series. This decreased slightly to average MAE of 0.176 using the gene distributions as a calibration objective during optimization (Additional file 6).

While the fit to the observed chemistry alone worsened slightly as a result of the calibration (ΔMAE_average_ = + 6.9e−3), the fit to observed gene profiles improved substantially (ΔMAE_average_ = − 5.4e−2). The majority of this improvement stemmed from a much better accordance between the calibrated model’s predictions of sulfur oxidation and sulfate reduction and the observed abundances of the *dsrAB* gene (ΔMAE = − 0.31).

**Fig. 3:**
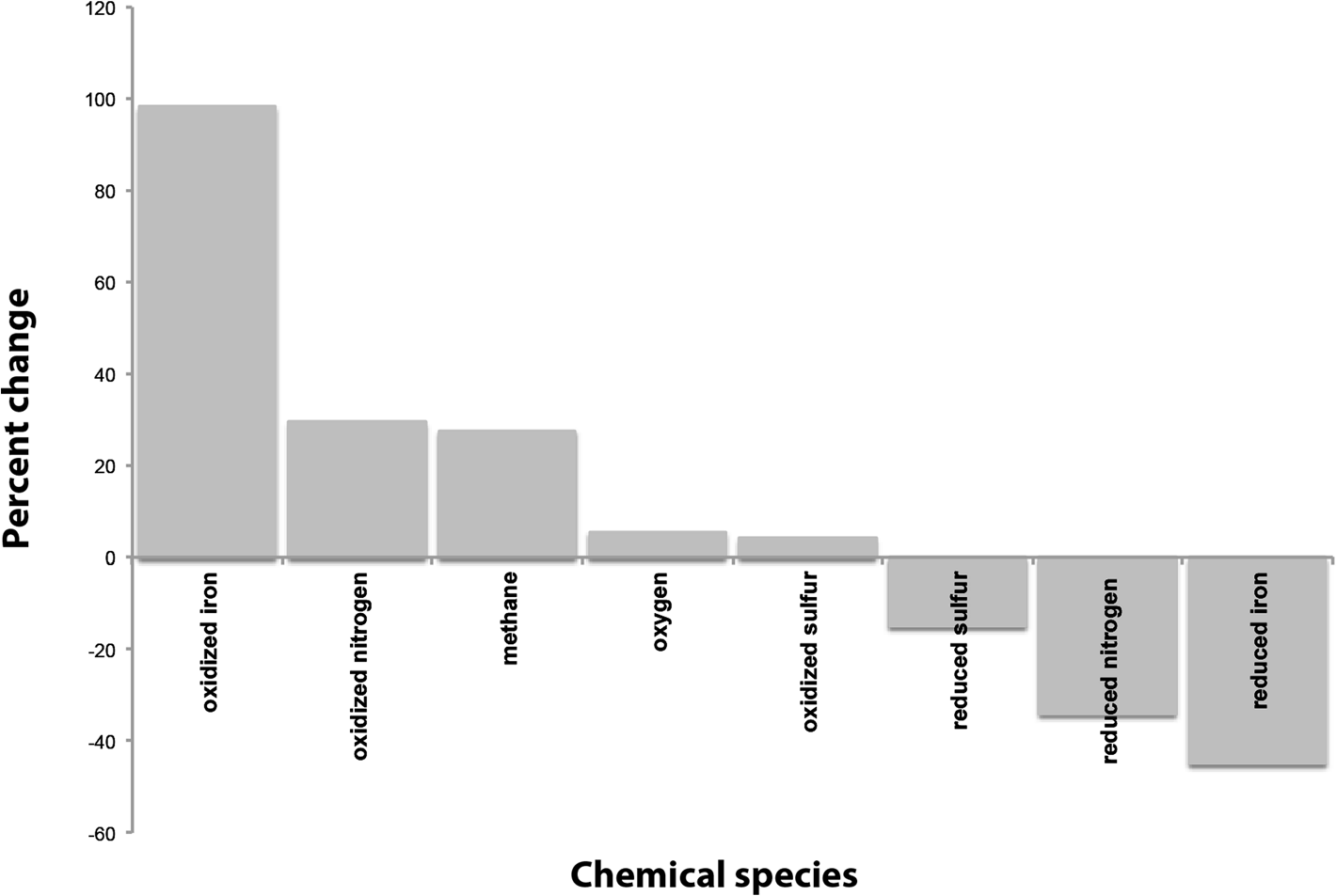
Percent change in modeled chemical species after removing denitrification coupled to methane and sulfur oxidation from the optimized model. After calibrating the model to match the chemical and gene distributions with the additional processes, denitrification coupled to methane and sulfur oxidation rate constants were set to zero, but all other parameters remained constant. Chemical concentrations were summed over all depths and time points. Removing these processes most substantially impacts iron speciation likely because of the competition with iron oxidation for nitrate

After having identified appropriate parameters for these additional processes, we could use the model to predict the impact of these processes on the ecosystem. Removing methane and sulfur oxidation with denitrification from the newly calibrated model substantially changes the predicted chemical composition of the lake (Fig. 3). The total amount of oxidized nitrogen in the lake increased when these processes were removed, as would be expected. However, the removal of these processes most significantly impacted predicted iron speciation in the lake. With the removal of these processes, the concentration of oxidized iron increased by over 98%, likely due to competition of these denitrification processes with nitrate-driven iron oxidation [32]. Across 10,000 randomly sampled values within the defined bounds of parameter space, the average percent change in oxidized iron was 402% (minimum 17%, maximum 2766%), suggesting that changes in oxidized iron concentrations when methane and sulfide oxidation on nitrate are removed are robust under various model configuration (Additional file 7). These results demonstrate that these processes could have a substantial impact on lake biogeochemistry if active. Further work is needed to determine whether these processes are active in the lake.

### Spatial and temporal dynamics of OTUs compared to model predictions

**Fig. 4:**
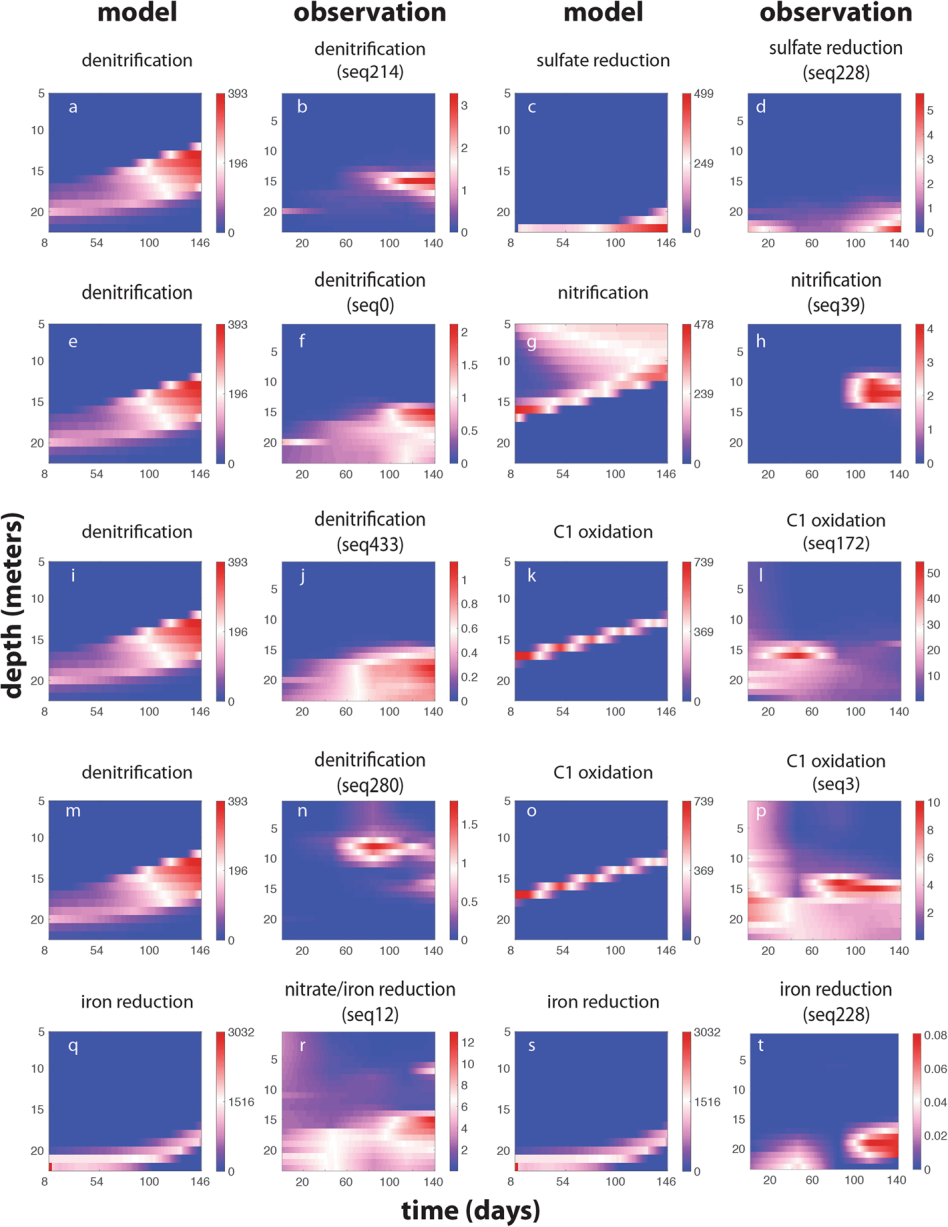
**a–t** Dynamics of populations capable of mediating the modeled processes. The spatiotemporal distribution of OTUs (second and fourth column) and associated processes predicted by the model (first and third column, respectively). Each panel has a its own key to the right of the graph indicating the color coding specific to each graph for the relative abundance (percent of total) of each OTU or rate (μM y^-1^) of each process. The model was not calibrated using OTU dynamics; thus, the relationship between the model and observations suggests that the availability of energy is an underlying factor driving the spatiotemporal dynamics of the most abundant and active microorganisms in the lake

The dynamics of populations could be influenced by many different factors, including the availability of energy for metabolic processes captured by the model. Although there is evidence that methane and sulfide oxidation coupled denitrification could be important processes in the lake, we continued with the previously published model configuration. In general, OTU dynamics were qualitatively similar to the dynamics predicted by the model for the process they were involved in (Fig. 4). The three of the four *Bacteroidetes* OTUs associated with heterotrophic denitrification (Fig. 4b, f, and j) had a distribution qualitatively similar to model predictions (Fig. 4a). Seq280 has a substantially different distribution from the other sequences and the modeled process (Fig. 4n). Iron-reducing OTUs similar to *Rhodoferax* (Fig. 4r) and *Geobacter* (Fig. 4t) had a distribution that was similar to iron reduction predictions (Fig. 4q, s), although *Rhodoferax* seemed to have a distribution influenced by denitrification, likely given the observed capability for nitrate reduction. The single sulfate-reducing population (Fig. 4d) has a distribution similar to sulfate reduction predicted by the model (Fig. 4c). The ammonia-oxidizing population (Fig. 4h) only matched the nitrification distribution for part of the season (Fig. 4g).

Methanotroph (Fig. 4l) and methylotrophs (Fig. 4p) displayed bloom-like dynamics distinct from the constant rate of methane oxidation predicted by the model (Fig. 4k and n). When these organisms did bloom, they were found at a depth that largely corresponded to the model prediction for methane metabolism. Shifts in the ratio of relative abundance of these C1-oxidizing populations from the amplicon data correspond to deviations between the model and observations for nitrate and iron (Additional file 8). Although these MAGs differ in the presence of methane monooxygenase (*pmoABC*) and *mxa* (methanol dehydrogenase) genes, they are both involved in metabolizing C1 compounds generated from methane. Interestingly, both nitrate and iron deviate from model predictions most dramatically during shifts in the ratio of these populations in the water column. Thus, their dynamics in the lake could represent shifts in the efficiency or use of nitrate as an electron acceptor in C1 compound oxidation. More work is needed to determine the cause of population shifts and their relationship to chemical deviations from the model.

## Discussion

This work provides insight into the relationship between microbial genes, populations, and a biogeochemical model of the processes they mediate to elucidate factors driving population dynamics and advance the use of population dynamics and MAG data in the development of predictive models. A biogeochemical model was used as our hypothesis of the factors influencing the dynamics of microbial populations mediating processes in the lake. It was also used to understand how adding or removing processes from the community would alter lake chemistry. We assumed that the presence of genes mediating the modeled processes would influence population dynamics and distribution; thus, the overall distribution of these genes would correspond to the modeled predictions. However, we also checked whether genes associated with different modeled processes co-occur within the MAG as a result of metabolic versatility, which would also influence gene distribution. Finally, we compared biogeochemical model predictions to the dynamics of key populations. We found that the dynamics of some microorganisms, such as those mediating denitrification, iron reduction, and sulfate reduction seem to be qualitatively captured by a dynamic model of energy availability. However, the model poorly captures the dynamics of key populations mediating methane oxidation, ammonia oxidation, and sulfur/iron oxidation.

There are multiple possible reasons for the lack of agreement between the diagnostic gene distributions and the associated processes predicted by the model. The model could be missing important processes, as suggested by the presence of sulfur-oxidizing denitrifiers. The original model only included sulfur oxidation with oxygen, shifting this process higher up in the water column than was observed for the genes. Pairing sulfur oxidation to denitrification would allow sulfur oxidizers to inhabit lower depths than pairing with oxygen since nitrate is found below where oxygen is depleted. Additionally, although the genes were present, they may not have been active. Genes could also exhibit substrate promiscuity, with genes assigned to one process mediating other processes. The ability of the well-known ferredoxin-nitrite reductase *nirA* to perform sulfite reduction is one such example [48]. An alternative explanation, especially applicable to bidirectional enzymes such as oxioreductases, is that the reverse reaction is occurring [49], as a result of the intracellular redox state or subtle sequence mutations affecting the reaction center. Our attempt to align genes to processes in the model relies on our ability to identify the genes involved. Thus, for environments such as this, where 63% of the genes are unknown or unclassified, many genes important to these processes could be unidentified.

The co-occurrence of autotrophic and denitrification genes in relatively abundant populations highlights the expanding view of nitrogen cycling. The original model coupled denitrification with primary carbon oxidation reactions [11], reflecting the idea that denitrification is largely associated with heterotrophic processes. However, this view has expanded as many researchers have identified the importance of denitrification in oxidation of methane [47], iron [50], and sulfur [42]. The combination of genes within autotrophic MAGs also supports this expanded role of denitrification within this system. Our original implementation of the biogeochemical model included iron oxidation coupled to denitrification based on previous observations from the lake [32, 50]. The similarity of a MAG to *Sideroxydans lithotrophicus* ES-1 supports this process. But the clustering of MAGs with other autotrophic denitrifiers and presence of *nosZ* genes suggests additional autotrophic denitrifying pathways should be considered.

Given the evidence for autotrophic processes competing for nitrate, these processes could directly impact greenhouse gases, nutrient cycling, and contaminant mobilization. Using the biogeochemical model, we demonstrate that competition for nitrate among autotrophic denitrifying populations could dramatically change iron speciation within the lake. Because of the importance of nitrate and iron in determining arsenic [32] and phosphate [33] speciation and mobility, these chemical species will likely be impacted as well. Additionally, nitrifiers have the denitrification gene *nirK*, providing it the potential for nitrifier denitrification, which can be a significant source of the potent greenhouse gas nitrous oxide in some ecosystems [51]. More work is needed to determine the rules governing these interactions and how the outcome of these competitive interactions between denitrifying populations could impact the overall chemistry of the lake.

While we could successfully identify MAGs linking functional capabilities to 16S rRNA gene sequences in our amplicon dataset, the development of more targeted methods to consistently associate function to 16S rRNA gene sequences would extend this analysis to more of the community. 16S rRNA gene sequencing can more efficiently provide population dynamics because many more samples can be sequenced with sufficient coverage as compared to shotgun metagenomics. Yet, limited functional information is available from 16S rRNA gene sequencing. Some currently available techniques, such as stable isotope analysis [52], or epicPCR [36], can help identify the 16S rRNA gene sequence associated with organisms containing specific capabilities or functional genes, but this targeted approach has limited use in creating a comprehensive understanding of community function and interactions. Although 16S rRNA gene sequences cannot always discriminate between closely related but functionally distinct organisms [53], the importance of these functional differences will depend on the process of interest. More phylogenetically constrained forms of energy conservation, like methane oxidation and sulfate reduction, will be less sensitive to this impact than processes such as denitrification [54]. More effective methods to tie 16S rRNA genes to associated function and MAGs would make the most efficient use of both types of sequencing and better inform dynamics and function.

The biogeochemical model serves multiple purposes in this analysis. First, it can be used to generate hypotheses about where processes are expected to occur given the current understanding of processes and the observed chemistry. It can then be used to compare to observations of genes and populations mediating the modeled processes. However, observations can help generate new ideas about the flow of energy and matter through the ecosystem, requiring updates to the model in an iterative approach. By comparing the previous model predictions with gene observations, we were able to identify major differences, which were supported by the MAG gene data. When we updated the model to reflect these observations, it allowed us to gain insight about potentially competing processes, which could substantially change iron chemistry in the lake. Finally, the model can be used to test unobserved conditions, such as the removal of methane and sulfide oxidation coupled to nitrate reduction from the ecosystem and observe the potential consequences. Not only can computational models facilitate the analysis of microbial communities, but it can also result in a usable product that may predict the response of the community to future environmental change scenarios.

While this analysis provides insight into potential capabilities and interactions between microorganisms, more work is needed to confirm activity and interactions. In general, the metabolic model and associated MAGs support the potential for these processes to occur given the chemical environment. Although we assume reproduction would concentrate cells at the various locations in the lake, the presence of microorganisms does not necessarily translate into metabolic activity. Microorganisms or their genes may not be active, even though there is an overall positive relationship in the water column between the presence (from DNA) and activity (from RNA) [17, 55]. Meta-transcriptomics could provide more insight into when and where organisms express the genes they carry. However, transcription of these key genes may only provide a snapshot of metabolic activities if they vary substantially between sampling periods [56]. Population abundances and their associated functional capabilities may integrate energy availability better than transcription over longer time-scales, especially if activity is intermittent within the specified time. On the other hand, population abundances are also determined by a complicated set of factors that might not be related to energy availability. Factors regulating population abundances may be influenced by other processes, such as immigration [57] and complex hydrodynamics, such as seiching [58]. However, within this lake, we have no evidence to suggest that hydrodynamic processes are the main drivers of the position of microbial populations.

## Conclusions

This analysis uses a combination of 16S rRNA gene surveys, metagenomic analysis, and biogeochemical modeling to gain insight into how energy availability shapes the distribution and dynamics of genes and microbial populations in the lake. Partial genome reconstruction through metagenomic binning provided insight into the capabilities of microbial populations mediating major biogeochemical cycles in the lake, including populations with genes supporting sulfur or methane oxidation coupled to denitrification. Modulating these processes in a biogeochemical model of the lake substantially changed predicted iron chemistry, which is recognized to impact both arsenic and phosphate mobilization. Secondary redox processes are not well captured by the model; thus, more work is needed to better understand the factors governing these processes. Overall, the relationship of the biogeochemical model predictions to the dynamics of populations mediating the primary redox processes identifies populations with dynamics largely controlled by energy availability as opposed to other factors. Additional observations of this ecosystem could shed light on how population dynamics are shaped by factors like phage predation, especially within methane oxidizers with potential for denitrification, which could impact ecosystem energy and material flow.

## Methods

### Sampling site and sampling protocol

Samples were collected from the middle of Upper Mystic Lake located in Medford, MA (42° 26.155′ N, 71° 08.961′ W), which has an approximate depth of 23 m. Samples were taken on March 26, May 10, June 17, July 17, and August 15, 2013, from the surface to 1 m off the bottom at 1 to 2 m intervals. For each sample, a peristaltic pump was used to pump 50 mL of water through a sterile 0.22-μm filter (Millipore), which was immediately placed on dry ice and stored at − 80 °C until DNA extraction. The filtrate was collected in a sterile 50-ml conical tube, placed immediate on dry ice, and stored at − 80 °C for anion analysis with ion chromatography. One milliliter of filtered and unfiltered water was added to 63 μl of concentrated hydrochloric acid for iron analysis with ferrozine [59]. An in situ probe was used to measure oxygen, temperature, and depth (Hydrolab MiniSonde). Water chemistry for the entire period and 16S rRNA gene libraries from DNA from Aug. 15, 2013, were used in a previous analysis [35].

### 16S rRNA gene library preparation and operational taxonomic unit (OTU) generation

DNA was extracted as previously described [35] using the PowerWater DNA extraction kit (MoBio Laboratories) following the manufacturer’s protocol with an alternative lysis step with the addition of 20 μl of proteinase K and incubation at 65 °C for 10 min before bead beating. 16S rRNA gene libraries of the V4 region of the 16S rRNA gene were generated using a two-step amplification method as described previously [35]. Universal primers targeting the region between the U515 and E786 positions were used (PE16S-V4-U515-F, 5′-ACACG ACGCT CTTCC GATCT YRYRG TGCCA GCMGC CGCGG TAA-3′ and PE16S-V4-E786-R, 5′-CGGCA TTCCT GCTGA ACCGC TCTTC CGATC TGGAC TACHV GGGTW TCTAA T-3′). The second set of primers contained Illumina adapter sequences and a 9-bp barcode for demultiplexing indicated by Ns (PE-III-PCR-F, 5′-AATGA TACGG CGACC ACCGA GATCT ACACT CTTTC CCTAC ACGAC GCTCT TCCGA TCT-3′ and PEIII-PCR-##-R, 5′-CAAGC AGAAG ACGGC ATACG AGATN NNNNN NNNCG GTCTC GGCAT TCCTG CTGAA CCGCT CTTCC GATCT-3′). Negative controls were also included to determine the contamination from the tubing and reagents. Positive control samples, including a mock community and replicate samples, were also included. Libraries were sequenced at Massachusetts Institute of Technology BioMicro Center (BMC) on an Illumina MiSeq with 250 bases for each of the forward and reverse reads and 8-base indexing read. One hundred two base pairs of the forward read were quality filtered, trimmed, and clustered into OTUs as previously described [35] using USEARCH [60], mothur [61] distribution-based clustering [62], and UCHIME [63] for quality filtering, sequence alignment, OTU calling, and chimera checking, respectively. Replicate samples were combined by their mean. Although the forward and reverse reads overlap in the amplicon library, 16S rRNA reads do not commonly overlap in the shotgun metagenomic library, described below. Since a direct comparison between 16S rRNA gene sequences generated in both datasets was used for quality control, the amplicon library sequence length was limited to 102 bps, a length used previously [62].

### Analysis of amplicon library control samples

To determine the influence of reagent contamination and methodological error on 16S rRNA gene sequence data, OTU tables and representative sequences were imported into QIIME2 [64] for diversity and principle coordinate analysis plots. The average read count for all negative samples was 1515 as compared to an average read count of 21,111 for positive samples and 62,838 for environmental samples. Negative samples were distinct from environmental samples (Additional file 9a), and negative samples typically contained a large proportion of OTUs classified as *Halomonas* while most of the other samples did not. Samples that did were discarded. Of the OTUs mentioned in the text, six were observed in the negative, but at much lower abundance (17 reads total) than in the environmental samples (1,194,496 reads total), suggesting contamination of negatives by samples, making it unlikely that these OTUs arose from contamination from reagents or other sources. Four mock community samples matched the known mock community sequences as expected, with little contamination. The sequenced reads matched the mock community with an average correlation coefficient of 0.77 and 0.89 with and without sequences with mismatches in the primer-binding site, respectively (Additional file 9b). Technical and biological replicates had an average Bray-Curtis distance of 0.19 (standard deviation +/− 0.09) compared to the average pairwise distance across the entire dataset of 0.69 (standard deviation +/− 0.2), suggesting that methodological variability was low compared to natural variability (Additional file 9d). Samples were processed and sequenced in random groups and did not cluster according to process group (Additional file 9c).

### Shotgun metagenomic library preparation and sequencing

Metagenomic libraries were made from samples taken at every other depth from 1 to 21 m, plus 20 and 22 m from the Aug. 15, 2013. Positive control libraries were made from *E. coli* (B strain) and *Microcystis aeruginosa* stock cultures (Carolina Biological Supply, Burlington, NC). Libraries were prepared using the Illumina Nextera XT kit (Illumina, San Diego, CA) according to the manufacturer’s protocol, except libraries were pooled manually. Libraries were sequenced at the Johns Hopkins University Genetic Resources Core Facility on an Illumina HiSeq 2500 (Illumina, San Diego, CA), generating 150 base pair paired-end sequences.

### Metagenomic binning

The requisite metadata information recommended by the Genomic Standards Consortium for metagenome-assembled genome [65] is shown in Additional file 1. The following approaches apply to all bins derived. Taxonomic classification was performed using megaBLAST [66] and the taxator-tk algorithm [67]. Reassembly of bins and initial co-assembly were performed using SPAdes 3.11 [68]. The initial assembly was completed in “metagenomic” mode, and reassembly was done for each bin in “careful” mode using the first pass contigs as “untrusted contigs.”

Contigs were binned according to their coverage and tetramer frequencies. A set of consensus bins were derived from the bins produced by the maxbin2 [69], metabat2 [70], and concoct [28] algorithms. Completeness and contamination assessment were performed using the lineage workflow in CheckM [71]. The preliminary bins mentioned above were those produced by *maxbin2*. Bins with < 70% completion and > 10% contamination were discarded. The bioinformatics pipeline from QC, to assembly, to binning, to refinement, to reassembly, and taxonomic classification was done within the metaWRAP software [72]. Prokka [73] was used to facilitate gene calling and preliminary annotations. Prodigal [74], barrnap, SSUsearch [75], and Aragorn v1.2 [76] were used to call open reading frames, ribosomal RNA, and transfer RNA. All protein sequences were generated using translation Table 11 within Prodigal.

### Binning fidelity

To investigate the consistency of the binning pipeline output as compared to contemporary alternatives [29], the published raw reads collected from a study in the Gulf of Mexico dead zone were processed in parallel with our samples. The bins produced by our pipeline were compared to metagenomic-assembled genomes uploaded and published to the IMG database. Using mash [77] to compare bins, the bins produced by *maxbin2* most closely matched those created in a previous analysis [29]. It is notable that even those bins that were manually refined into multiple smaller bins and presented as distinct organisms (43–1/2 and 45–1/2) were matched to unique bins produced by *maxbin2*.

Mash was used to determine the closest matches between bin sets [77]. Shared hashes are the units of homology produced by mash. Despite having greater sensitivity to mismatches and less accuracy than alignment-based comparisons, it was suitable for rapid similarity assessment. The degree of “concentration” or “specificity” of a match was derived from these data and was calculated as the ratio of shared hashes between the query genome and the single best reference genome, divided by the sum of hashes shared with all references. This value was calculated for members of a match to determine the degree of dispersion of a given MAG across the entire other bin set (Additional file 10). After establishing 1 to 1 pairing between MAG sets, Mummer was used to perform genome-to-genome alignments [78]. The “--mum” tag was used to ensure only unique alignments on both reference and query were produced. Alignments were filtered to only include regions with longer than 1 kbp of homology. The genome size and the fraction of the genome aligned for both members of a match are shown in the file mentioned above.

### Identification of genes involved in modeled metabolic processes

Protein annotations were conducted using KEGG BlastKOALA and GhostKOALA annotation web service [79]. Any classifications with a GHOSTX score below 100 were removed, as previously recommended [80]. C-type-cytochromes, involved in extracellular electron transfer to iron in *Geobacter* [81] and *Rhodoferax* [82], were identified by BLASTP and TBLASTX [83], respectively, with *e* value cutoff of 1E−190.

### Functional gene distribution and corresponding modeled processes

The abundance of select genes was annotated with biogeochemical process-related KEGG Ids. Salmon was used to determine the relative abundances of these sequences in each library [84]. The nucleotide sequence of each gene was bookended by 50–100 bp on each end to ensure that near identical gene sequences on distinct contigs were not conflated. Uniqueness was confirmed during indexing, as Salmon detects and removes duplicates prior to mapping.

Gene abundances values produced by Salmon are presented in sample-normalized units (i.e., copies per million). They were then L1 normalized by gene. These steps were taken as an alternative to normalizing according to average single-copy gene abundances [85]. The rationale for this approach stems from the fact that uncultivable organisms are not ensured to contain single copies of all single-copy genes. Furthermore, the ratio of binned to unbinned nucleotides is approximately 3:4 in our analysis. The majority of these unbinned contigs were contained in the 182 bins that were discarded based on the absence of > 30% of their single-copy genes. Taken together, these imply that the average single-copy gene abundance will likely underestimate the contribution of functional genes from unbinned fragments.

Protein annotations do not always correspond to a unique modeled process; thus, multiple modeled processes were compared to the gene distribution during model comparison and calibration. For these processes, the sum of all possible modeled reactions mediated by the gene was normalized and compared to the gene distribution. Ammonia oxidation, methane oxidation with oxygen, and methane oxidation with sulfate were used in the comparison with *pmoABC* genes, as these genes can mediate either methane or ammonia oxidation [86]. Sulfur oxidation and sulfate reduction were used in the comparison with *dsrAB* genes, as these could be involved in either sulfate reduction or sulfur oxidation [87]. Heterotrophic and autotrophic denitrification was used in the comparison with *nosDZ*. Methane oxidation with oxygen and sulfate were used in the comparison with *mxaCGKL*. Nitrification was represented with *hoa* and iron reduction with *Rhodoferax* and *Geobacter* iron reduction genes, matched by BLAST [83].

### Characterizing metabolic capabilities of metagenome-assembled genomes

Metabolic capabilities of MAGs were determined from gene content similarity to KEGG genomes with known functions and the presence of key genes and pathways within the MAG. KEGG ids extracted from the MGENE set associated with each KEGG genome (5647 total comparisons) were added to a matrix of all MAG KEGG ids individually. Normalization and complete linkage hierarchical clustering using Spearman Rank correlation placed the KEGG genome within the MAG tree using Cluster 3.0 [88]. The functional capabilities of up to four of the closest KEGG genomes were used to assess MAG functional capabilities. Additionally, the presence of specific genes within the MAG was used as validation for specific functional capabilities as described above for methane and ammonia oxidation (*pmoABC*; K10944, K10945, and K10946), nitrification (*hao*; K10535), methane oxidation (*mxaCGKL*; K16255, K16257, K16258, K16259), sulfur cycling (*dsrAB*; K11180 and K11181), denitrification (*nosDZ*; K07218, K00376), iron reduction (*Geobacter* and *Rhodoferax* genes), or carbon fixation (*rbcSL*; K01601, K01602).

### OTUs matching metagenomic bins

Both OTUs derived from shotgun libraries, as well as those observed in amplicon libraries were used assigned 16S rRNA gene sequences to bins based on similarities in abundance and taxonomic classification. The top 500 amplicon OTUs by the sum of their abundances in comparable samples were used in the analysis. The 11 overlapping samples in both the metagenomic shotgun dataset and the amplicon library dataset were used in the comparison. Abundances were normalized using the L1 norm along both axes, within samples first and then within each amplicon/bin.

Raw abundance (*A*) of a bin in a given sample *j* were calculated within metaWRAP using the following equation:

$$ {A}_j=\frac{\sum_{i=1}^N{l}_i{c}_{ij}}{\sum_{i=1}^N{l}_i} $$

The product of the length of contig (*l*_*i*_) and its coverage (*c*_*ij*_) in sample *j* was summed across all *N* contigs assigned to the bin and then divided by total length of the bin genome. These raw values were divided by the number of reads in each library before following the same normalization steps applied to OTU abundances.

OTU taxonomy was assigned using the RDP classifier and the 16S rRNA gene Training Set 16 [89]. Confidence scores below 50% and *Incertae Sedis* classifications were removed from the “fixed rank” output. Each level in the hierarchy from kingdom to genus was treated as an independent feature to match the format of the taxonomic assignments produced for each bin. Pairwise Euclidean distances between abundance vectors for bins, and OTUs were calculated and seen to range from 0 to 1 on L1 normalized vectors. The fraction of matching taxonomic hierarchical levels (using the bin taxonomy as the denominator) was then subtracted from the distance to produce a combined distance metric ranging from 1 to −1, where the latter represents a perfect match. Eight bins were used as a positive control for the matching procedure, as five bins contained assembled 16S rRNA genes and three bins contained known positive control organisms. The tolerance was trained on these bins such that the minimum distance needed to capture all the correct assignments was accepted. This tolerance value was observed to be different for the full time series of amplicon libraries (0.0251), as compared to shotgun library data (0.176).

### Incorporating additional processes into the biogeochemical model

The biogeochemical model is a conceptual representation of the lake, modeled as previously described [35]. Two processes were added to the biogeochemical model to reflect the information obtained from the metagenomic bins. Denitrification by sulfur-oxidizing autotrophs was added assuming the stoichiometry [45]:

$$ 5\ {\mathrm{H}\mathrm{S}}^{-}+8\ {{\mathrm{N}\mathrm{O}}_3}^{-}+3\ {\mathrm{H}}^{+}\to 5\ {{\mathrm{SO}}_4}^{2-}+4\ {\mathrm{N}}_2+4\ {\mathrm{H}}_2\mathrm{O} $$

Denitrification by methane-oxidizing autotrophs was added assuming the stoichiometry [90]:

$$ 5\ {\mathrm{CH}}_4+8\ {{\mathrm{N}\mathrm{O}}_3}^{-}+8\ {\mathrm{H}}^{+}\to 5\ {\mathrm{CO}}_2+4\ {\mathrm{N}}_2+14\ {\mathrm{H}}_2\mathrm{O} $$

Finally, a precipitation constant for reduced sulfur was added to simulate the likely formation of iron mono-sulfide, pyrite, or organic sulfur precipitates [91, 92].

### Gene- and concentration-driven model calibration

Model parameters were calibrated to fit the gene and chemical observations. The calibrated parameters include rate constants, precipitation constants, and scalars applied to initial concentration profiles of unmeasured chemicals. Calibrated parameters were constrained using existing literature evidence when available.

Model calibration was achieved using a stochastic, sequential, and bounded search algorithm (https://github.com/spacocha/MWMW/blob/master/Scripts/calibration_script.py). The algorithm sequentially optimizes and fixes the value of each parameter. For each parameter, a fixed number (100–1000) of simulations is performed. Parameter values are randomly sampled from Gaussian distributions whose boundaries are the limits drawn from the literature. At the end of an iteration, an *F* test is performed with the sampled parameter values as features and the observed fit metric as a response. The parameter with the lowest *p* value (i.e., the most sensitive) is fixed at its best-fitting value. Next, sampling distributions for the values of the unfixed parameters in the next set of simulations are constructed. Linear regression models are fit individually using the performance metric to reset the centers of the Gaussian distributions for unfixed parameters. The distance between the current and previous centers for these unfixed parameters is used to define the shape of the Gaussian. A burn-in iteration in which no parameter is fixed is used to calculate the initial centers for subsequent iterations steps. Once all parameters are fixed, additional polishing is achieved using the L-BFGS-B algorithm [93] initiated at the point of convergence.

The performance metric utilized was the average mean absolute error between observed and modeled concentrations and process rates. Observed concentrations of some oxidized species were used directly for this purpose, and the average normalized abundances of specific gene sets were used as a proxy for microbial process. A list of parameters, calibration search ranges, and final value selections can be found in the script repository (https://github.com/spacocha/MWMW/tree/master/Data/calibration_data).

## Additional files

Additional file 1: MAG quality stats. Table of the percent completion, percent contamination, GC%, taxonomic lineage, and other genome statistics for each MAG. (XLSX 24 kb)

Additional file 2: Clustering of MAGs with KEGG genome by shared gene content. Heat map and hierarchical tree structures associated with clustering of MAGs to representative KEGG genomes, and genes indicated by KEGG Ids for a subset of the genes used in cluster analysis. (TIF 1896 kb)

Additional file 3: Top 4 KEGG Genome matches for MAGs and associated biogeochemical processes. Table of KEGG genomes that have the lowest distance to each MAG. (XLSX 23 kb)

Additional file 4: MAG gene list. A table of the presence of genes associated with specific biogeochemical processes within MAGs. (XLSX 66 kb)

Additional file 5: Sub-OTU level diversity within *Geobacter* OTU. A graph of the longer 250 bp Geobacter OTUs within the water column on 8/12/13 as compared to MAGs that contain the most Geobacter iron-reducing genes. (PDF 28 kb)

Additional file 6: Comparison of model fit and optimized parameters before and after calibration. Fit metrics and model parameters used for the previous model and calibrated model. (XLSX 45 kb)

Additional file 7: Results from 10,000 randomly sampled values within the defined bounds of parameter space. Graph of average values for each chemical species, percent change with and without sulfur, and methane oxidation with denitrification, including average and standard deviations. (XLSX 993 kb)

Additional file 8: Deviation between modeled and observed concentration of nitrate and iron correspond to shifts in methane-oxidizing populations. A graph showing the deviation in modeled values for iron and nitrate (left *y*-axis) and the corresponding ratio (right *y*-axis) of C1 oxidizers *Methyloterna versitalis* (seq3) and *Methylomicrobium alcaliphilum* (seq172) over the time series. (PDF 32 kb)

Additional file 9: Control analysis of Mystic Lake and control samples. a.) PCoA plot of samples colored according to depth name, with surface and reagent negative control samples in blue, and environmental samples in red with Bray-Curtis as distance metric. This demonstrates that negative samples are distinct from environmental samples. b.) Comparison between the observed read count and input template concentration for OTU sequences matching the mock community sequences (primer site exact matches only) for one representative mock community. This demonstrates that input template concentration largely explains the resulting read count distribution. c.) PCoA plot of samples colored according to process group (various colors) with Bray-Curtis as distance metric. This demonstrates that sample do not cluster according to process group. d.) Comparison of the average and standard deviation of Bray-Curtis distance metric for technical and biological replicates as compared to the average and standard deviation of all samples. This demonstrates that most of the variability in the dataset is not due to methodological errors. (TIF 1362 kb)

Additional file 10: Comparison of control bins to previously published MAGs. Statistics of the comparison between previously published MAGs and our assembly with metaWRAP, which shows a high degree of similarity, demonstrating that our method of assembly and binning is similar to previously published metagenomic analyses. (TSV 1 kb)

## Abbreviations

HMM: Hidden Markov Model
KEGG: Kyoto Encyclopedia of Genes and Genomes
MAE: Mean absolute error
MAG: Metagenome-assembled genome
OTU: Operational taxonomic unit
Redox: Oxidation-reduction

## References

[1] U.S. Environmental Protection Agency, O.o.W (2004). National water quality inventory: report to congress, 2004 reporting cycle.

[2] Limburg KE, Breitburg D, Levin LA (2017). Ocean deoxygenation—a climate-related problem. Front Ecol Environ.

[3] McCrackin ML (2017). Recovery of lakes and coastal marine ecosystems from eutrophication: a global meta-analysis. Limnol Oceanogr.

[4] Lloyd JR, Lovley DR (2001). Microbial detoxification of metals and radionuclides. Curr Opin Biotech.

[5] Cullen WR, Reimer KJ (1989). Arsenic speciation in the environment. Chem Rev.

[6] Mortimer CH (1941). The exchange of dissolved substances between mud and water in lakes. J Ecol.

[7] Hupfer M, Lewandowski J (2008). Oxygen controls the phosphorus release from lake sediments—a long-lasting paradigm in limnology. Int Rev Hydrobiol.

[8] Smith SV, Hollibaugh JT (1989). Carbon-controlled nitrogen cycling in a marine macrocosm—an ecosystem-scale model for managing cultural eutrophication. Mar Ecol Prog Ser.

[9] Conley DJ (2009). Hypoxia-related processes in the Baltic Sea. Environ Sci Technol.

[10] Dickinson RE, Cicerone RJ (1986). Future global warming from atmospheric trace gases. Nature.

[11] Hunter KS, Wang YF, Van Cappellen P (1998). Kinetic modeling of microbially-driven redox chemistry of subsurface environments: coupling transport, microbial metabolism and geochemistry. J Hydrol.

[12] Nannipieri P (2003). Microbial diversity and soil functions. Eur J Soil Sci.

[13] Burke C (2011). Bacterial community assembly based on functional genes rather than species. P Natl Acad Sci USA..

[14] Fan L (2012). Functional equivalence and evolutionary convergence in complex communities of microbial sponge symbionts. P Natl Acad Sci USA..

[15] Huttenhower C (2012). Structure, function and diversity of the healthy human microbiome. Nature.

[16] Reed DC (2014). Gene-centric approach to integrating environmental genomics and biogeochemical models. P Natl Acad Sci USA..

[17] Louca S (2016). Integrating biogeochemistry with multiomic sequence information in a model oxygen minimum zone. P Natl Acad Sci USA..

[18] Hooper DU (2005). Effects of biodiversity on ecosystem functioning: a consensus of current knowledge. Ecol Monogr.

[19] Vitousek PM, Walker LR (1989). Biological invasion by Myrica-Faya in Hawaii—plant demography, nitrogen-fixation, ecosystem effects. Ecol Monogr.

[20] Beschta RL, Ripple WJ (2009). Large predators and trophic cascades in terrestrial ecosystems of the western United States. Biol Conserv.

[21] Rousseaux CS, Gregg WW (2014). Interannual variation in phytoplankton primary production at a global scale. Remote Sens-Basel.

[22] Zhuang K (2011). Genome-scale dynamic modeling of the competition between Rhodoferax and Geobacter in anoxic subsurface environments. Isme J.

[23] Canfield DE, Kristensen E, Thamdrup B. Heterotrophic carbon metabolism. Advances in Marine Biology. 2005;48:129–66. 10.1016/S0065-2881(05)48017-7 15797449

[24] Boetius A (2000). A marine microbial consortium apparently mediating anaerobic oxidation of methane. Nature.

[25] Orphan VJ (2009). Methods for unveiling cryptic microbial partnerships in nature. Curr Opin Microbiol.

[26] Dumont MG (2011). DNA-, rRNA- and mRNA-based stable isotope probing of aerobic methanotrophs in lake sediment. Environ Microbiol.

[27] McGlynn SE (2015). Single cell activity reveals direct electron transfer in methanotrophic consortia. Nature.

[28] Alneberg J (2014). Binning metagenomic contigs by coverage and composition. Nat Methods.

[29] Thrash JC (2017). Metabolic roles of uncultivated bacterioplankton lineages in the Northern Gulf of Mexico “dead zone”. Mbio.

[30] Faust Karoline, Raes Jeroen (2012). Microbial interactions: from networks to models. Nature Reviews Microbiology.

[31] Trosvik P, de Muinck EJ, Stenseth NC (2015). Biotic interactions and temporal dynamics of the human gastrointestinal microbiota. Isme J.

[32] Senn DB, Hemond HF (2002). Nitrate controls on iron and arsenic in an urban lake. Science.

[33] Hemond HF, Lin K (2010). Nitrate suppresses internal phosphorus loading in an eutrophic lake. Water Res.

[34] Varadharajan Charuleka, Hemond Harold F. (2012). Time-series analysis of high-resolution ebullition fluxes from a stratified, freshwater lake. Journal of Geophysical Research: Biogeosciences.

[35] Preheim SP (2016). Surveys, simulation and single-cell assays relate function and phylogeny in a lake ecosystem. Nature Microbiology.

[36] Spencer Sarah J, Tamminen Manu V, Preheim Sarah P, Guo Mira T, Briggs Adrian W, Brito Ilana L, A Weitz David, Pitkänen Leena K, Vigneault Francois, Virta Marko PJuhani, Alm Eric J (2015). Massively parallel sequencing of single cells by epicPCR links functional genes with phylogenetic markers. The ISME Journal.

[37] Noble PA, Citek RW, Ogunseitan OA (1998). Tetranucleotide frequencies in microbial genomes. Electrophoresis.

[38] Bock E (1995). Nitrogen loss caused by denitrifying Nitrosomonas cells using ammonium or hydrogen as electron-donors and nitrite as electron-acceptor. Arch Microbiol.

[39] Schmidt I, van Spanning RJM, Jetten MSM (2004). Denitrification and ammonia oxidation by Nitrosomonas europaea wild-type, and NirK- and NorB-deficient mutants. Microbiol-Sgm..

[40] Emerson D, et al. Comparative genomics of freshwater Fe-oxidizing bacteria: implications for physiology, ecology, and systematics. Front Microbiol; 2013. 10.3389/fmicb.2013.00254. 10.3389/fmicb.2013.00254 PMC3770913 24062729

[41] Kojima H, Fukui M (2011). Sulfuritalea hydrogenivorans gen. nov., sp nov., a facultative autotroph isolated from a freshwater lake. Int J Syst Evol Micr..

[42] Kojima H, Fukui M (2010). Sulfuricella denitrificans gen. nov., sp nov., a sulfur-oxidizing autotroph isolated from a freshwater lake. Int J Syst Evol Micr.

[43] Vuilleumier S (2012). Genome sequence of the Haloalkaliphilic methanotrophic bacterium Methylomicrobium alcaliphilum 20Z. J Bacteriol.

[44] Kits KD, et al. Diverse electron sources support denitrification under hypoxia in the obligate methanotroph Methylomicrobium album strain BG8. Front Microbiol; 2015. 10.3389/fmicb.2015.01072. 10.3389/fmicb.2015.01072 PMC4594100 26500622

[45] Burgin AJ (2012). Denitrification by sulfur-oxidizing bacteria in a eutrophic lake. Aquat Microb Ecol.

[46] Al Azhar M (2014). A model-based insight into the coupling of nitrogen and sulfur cycles in a coastal upwelling system. J Geophys Res-Biogeo.

[47] Deutzmann JS (2014). Anaerobic methane oxidation coupled to denitrification is the dominant methane sink in a deep lake. P Natl Acad Sci USA..

[48] Maia LB, Moura JJG (2014). How biology handles nitrite. Chem Rev.

[49] Thorup C, et al. Disguised as a sulfate reducer: growth of the deltaproteobacterium Desulfurivibrio alkaliphilus by sulfide oxidation with nitrate. MBio; 2017. 10.1128/mBio.00671-17. 10.1128/mBio.00671-17 PMC5516251 28720728

[50] Senn DB. Coupled arsenic, iron, and nitrogen cycling in arsenic-contaminated Upper Mystic Lake; 2001. http://hdl.handle.net/1721.1/8750. Accessed 12 Sep 2018.

[51] Kool DM (2011). Nitrifier denitrification as a distinct and significant source of nitrous oxide from soil. Soil Biol Biochem.

[52] Radajewski S (2000). Stable-isotope probing as a tool in microbial ecology. Nature.

[53] Nelson WC (2016). Identification and resolution of microdiversity through metagenomic sequencing of parallel consortia. Appl Environ Microb..

[54] Martiny Adam C, Treseder Kathleen, Pusch Gordon (2012). Phylogenetic conservatism of functional traits in microorganisms. The ISME Journal.

[55] Hunt DE (2013). Relationship between abundance and specific activity of bacterioplankton in open ocean surface waters. Appl Environ Microb.

[56] Ottesen EA (2013). Pattern and synchrony of gene expression among sympatric marine microbial populations. P Natl Acad Sci USA.

[57] Nemergut DR (2013). Patterns and processes of microbial community assembly. Microbiol Mol Biol R.

[58] Cuypers Y (2011). Impact of internal waves on the spatial distribution of Planktothrix rubescens (cyanobacteria) in an alpine lake. Isme J..

[59] Stookey LL (1970). Ferrozine—a new spectrophotometric reagent for iron. Anal Chem.

[60] Edgar RC (2013). UPARSE: highly accurate OTU sequences from microbial amplicon reads. Nat Methods.

[61] Schloss PD (2009). Introducing mothur: open-source, platform-independent, community-supported software for describing and comparing microbial communities. Appl Environ Microb..

[62] Preheim SP (2013). Distribution-based clustering: using ecology to refine the operational taxonomic unit. Appl Environ Microb..

[63] Edgar Robert C., Haas Brian J., Clemente Jose C., Quince Christopher, Knight Rob (2011). UCHIME improves sensitivity and speed of chimera detection. Bioinformatics.

[64] Caporaso JG (2010). QIIME allows analysis of high-throughput community sequencing data. Nat Methods.

[65] Bowers RM (2017). Minimum information about a single amplified genome (MISAG) and a metagenome-assembled genome (MIMAG) of bacteria and archaea. Nat Biotechnol.

[66] Morgulis A (2008). Database indexing for production MegaBLAST searches. Bioinformatics.

[67] Droge J, Gregor I, McHardy AC (2015). Taxator-tk: precise taxonomic assignment of metagenomes by fast approximation of evolutionary neighborhoods. Bioinformatics.

[68] Bankevich A (2012). SPAdes: a new genome assembly algorithm and its applications to single-cell sequencing. J Comput Biol.

[69] Wu YW, Simmons BA, Singer SW (2016). MaxBin 2.0: an automated binning algorithm to recover genomes from multiple metagenomic datasets. Bioinformatics.

[70] Kang Dongwan D., Froula Jeff, Egan Rob, Wang Zhong (2015). MetaBAT, an efficient tool for accurately reconstructing single genomes from complex microbial communities. PeerJ.

[71] Parks DH (2015). CheckM: assessing the quality of microbial genomes recovered from isolates, single cells, and metagenomes. Genome Res.

[72] Uritskiy GV, DiRuggiero J, Taylor J. MetaWRAP—a flexible pipeline for genome-resolved metagenomic data analysis. 2018. 10.1101/277442:. 10.1186/s40168-018-0541-1 PMC6138922 30219103

[73] Seemann T (2014). Prokka: rapid prokaryotic genome annotation. Bioinformatics.

[74] Hyatt Doug, Chen Gwo-Liang, LoCascio Philip F, Land Miriam L, Larimer Frank W, Hauser Loren J (2010). Prodigal: prokaryotic gene recognition and translation initiation site identification. BMC Bioinformatics.

[75] Guo Jiarong, Cole James R., Zhang Qingpeng, Brown C. Titus, Tiedje James M. (2015). Microbial Community Analysis with Ribosomal Gene Fragments from Shotgun Metagenomes. Applied and Environmental Microbiology.

[76] Laslett D, Canback B (2004). ARAGORN, a program to detect tRNA genes and tmRNA genes in nucleotide sequences. Nucleic Acids Res.

[77] Ondov BD, et al. Mash: fast genome and metagenome distance estimation using MinHash. Genome Biol; 2016. 10.1186/s13059-016-0997-x. 10.1186/s13059-016-0997-x PMC4915045 27323842

[78] Marçais Guillaume, Delcher Arthur L., Phillippy Adam M., Coston Rachel, Salzberg Steven L., Zimin Aleksey (2018). MUMmer4: A fast and versatile genome alignment system. PLOS Computational Biology.

[79] Kanehisa M, Sato Y, Morishima K (2016). BlastKOALA and GhostKOALA: KEGG tools for functional characterization of genome and metagenome sequences. J Mol Biol.

[80] Raethong Nachon, Wong-ekkabut Jirasak, Laoteng Kobkul, Vongsangnak Wanwipa (2016). Sequence- and Structure-Based Functional Annotation and Assessment of Metabolic Transporters inAspergillus oryzae: A Representative Case Study. BioMed Research International.

[81] Butler Jessica E, Young Nelson D, Lovley Derek R (2010). Evolution of electron transfer out of the cell: comparative genomics of six Geobacter genomes. BMC Genomics.

[82] Risso C (2009). Genome-scale comparison and constraint-based metabolic reconstruction of the facultative anaerobic Fe(III)-reducer Rhodoferax ferrireducens. BMC Genomics.

[83] Altschul SF (1990). Basic local alignment search tool. J Mol Biol.

[84] Patro R (2017). Salmon provides fast and bias-aware quantification of transcript expression. Nat Methods.

[85] Rinke C (2013). Insights into the phylogeny and coding potential of microbial dark matter. Nature.

[86] Holmes AJ (1995). Evidence that particulate methane monooxygenase and ammonia monooxygenase may be evolutionarily related. FEMS Microbiol Lett.

[87] Pott AS, Dahl C (1998). Sirohaem sulfite reductase and other proteins encoded by genes at the dsr locus of Chromatium vinosum are involved in the oxidation of intracellular sulfur. Microbiol-Sgm.

[88] de Hoon MJL (2004). Open source clustering software. Bioinformatics.

[89] Cole JR (2014). Ribosomal database project: data and tools for high throughput rRNA analysis. Nucleic Acids Res.

[90] Raghoebarsing AA (2006). A microbial consortium couples anaerobic methane oxidation to denitrification. Nature.

[91] Lyons TW (1997). Sulfur isotopic trends and pathways of iron sulfide formation in upper Holocene sediments of the anoxic Black Sea. Geochim Cosmochim Ac..

[92] Raven Morgan Reed, Sessions Alex L., Adkins Jess F., Thunell Robert C. (2016). Rapid organic matter sulfurization in sinking particles from the Cariaco Basin water column. Geochimica et Cosmochimica Acta.

[93] Byrd RH (1995). A limited memory algorithm for bound constrained optimization. SIAM J Sci Comput.

