# Supplementary figures and images for: Dynamics of microbial populations mediating biogeochemical cycling in a freshwater lake
Source: Microbiome. 2018 Sep 18;6:165. doi: 10.1186/s40168-018-0556-7 (PMC6145348; doi:10.1186/s40168-018-0556-7)

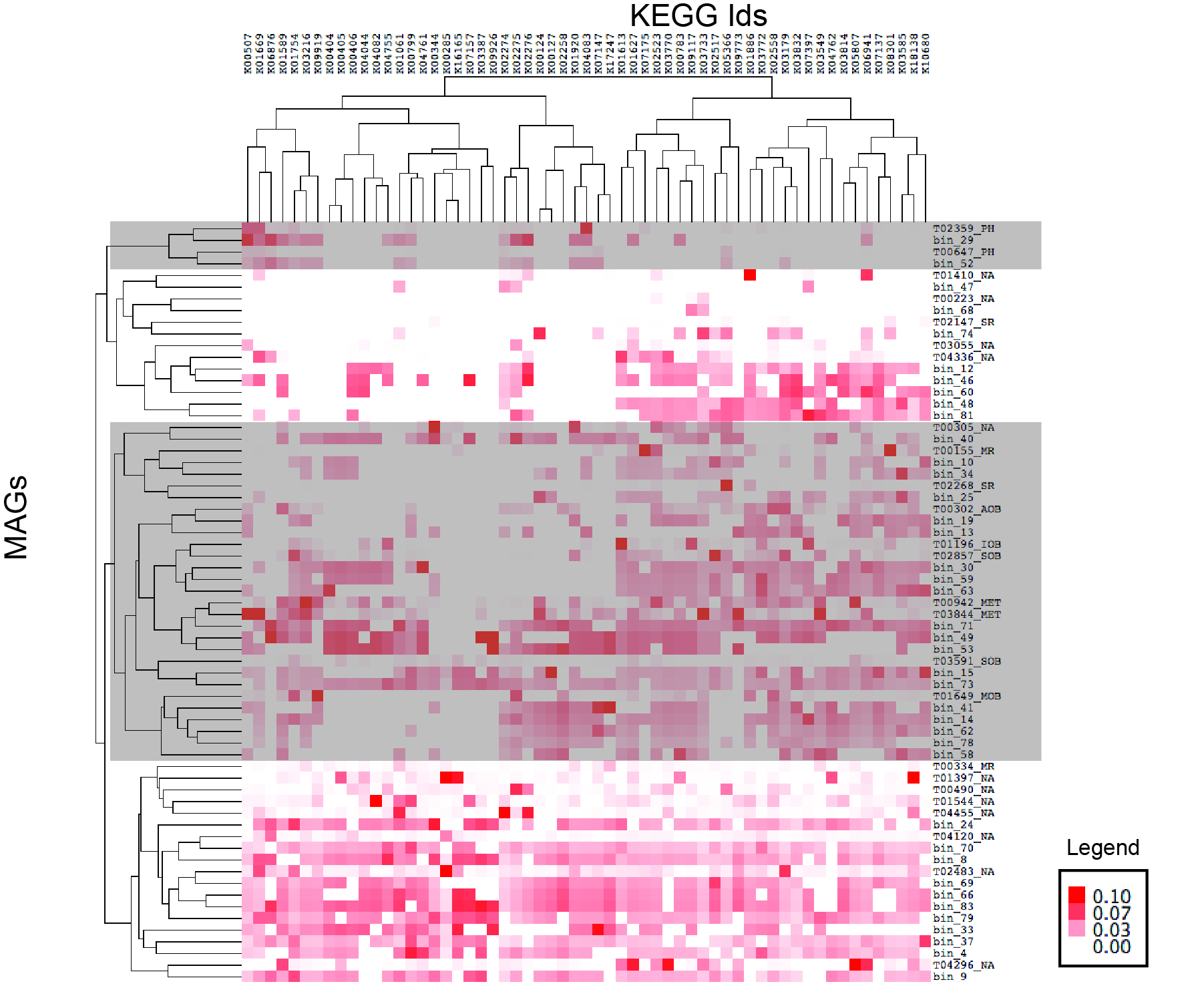

Supplement: Supplementary file 2 — Clustering of MAGs with KEGG genome by shared gene content. Heat map and hierarchical tree structures associated with clustering of MAGs to representative KEGG genomes, and genes indicated by KEGG Ids for a subset of the genes used in cluster analysis. (TIF 1896 kb) [file 40168_2018_556_MOESM2_ESM.tif]

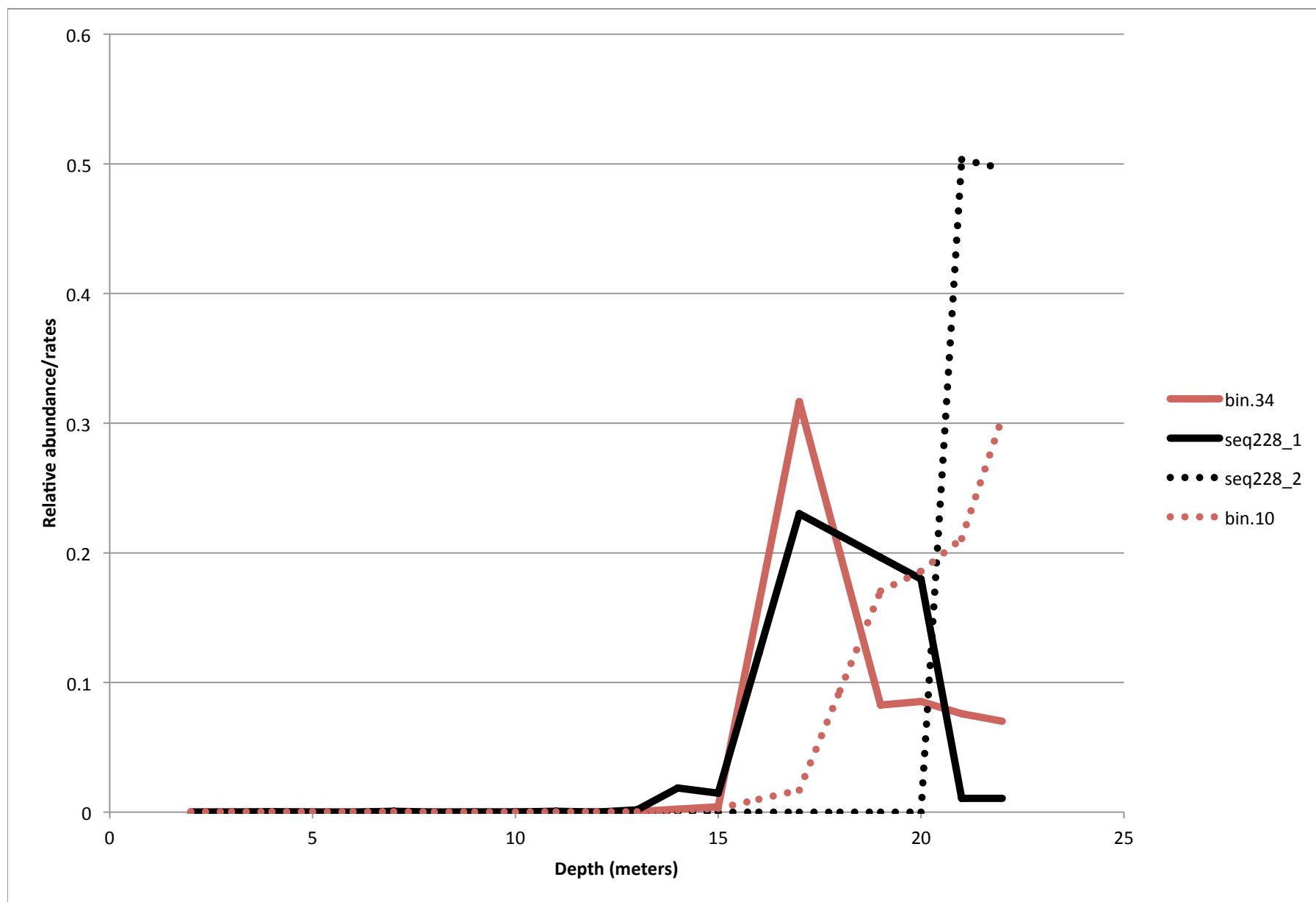

Supplement: Supplementary file 5 — Sub-OTU level diversity within Geobacter OTU. A graph of the longer 250 bp Geobacter OTUs within the water column on 8/12/13 as compared to MAGs that contain the most Geobacter iron-reducing genes. (PDF 28 kb) [file 40168_2018_556_MOESM5_ESM.pdf]

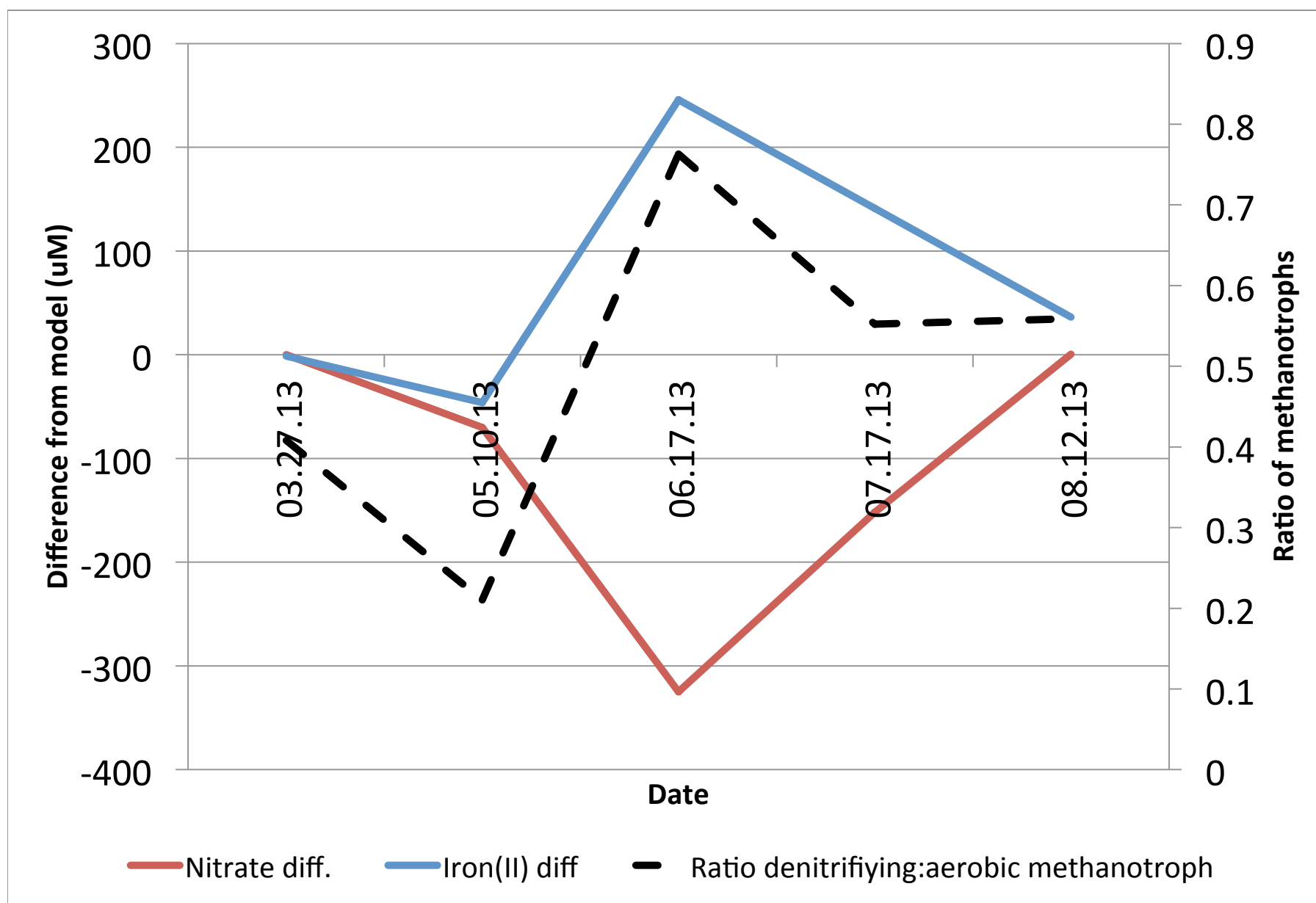

Supplement: Supplementary file 8 — Deviation between modeled and observed concentration of nitrate and iron correspond to shifts in methane-oxidizing populations. A graph showing the deviation in modeled values for iron and nitrate (left y-axis) and the corresponding ratio (right y-axis) of C1 oxidizers Methyloterna versitalis (seq3) and Methylomicrobium alcaliphilum (seq172) over the time series. (PDF 32 kb) [file 40168_2018_556_MOESM8_ESM.pdf]

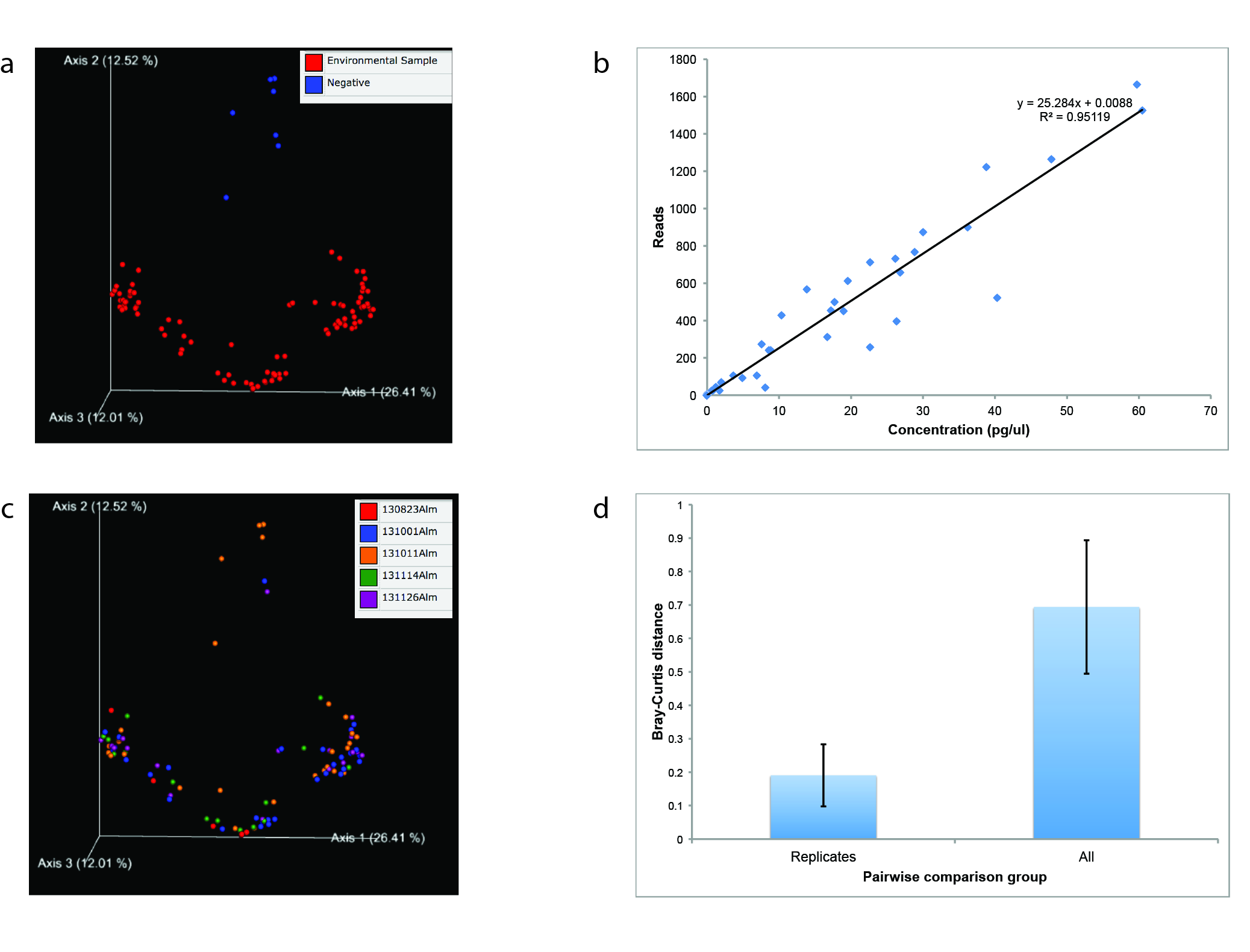

Supplement: Supplementary file 9 — Control analysis of Mystic Lake and control samples. a.) PCoA plot of samples colored according to depth name, with surface and reagent negative control samples in blue, and environmental samples in red with Bray-Curtis as distance metric. This demonstrates that negative samples are distinct from environmental samples. b.) Comparison between the observed read count and input template concentration for OTU sequences matching the mock community sequences (primer site exact matches only) for one representative mock community. This demonstrates that input template concentration largely explains the resulting read count distribution. c.) PCoA plot of samples colored according to process group (various colors) with Bray-Curtis as distance metric. This demonstrates that sample do not cluster according to process group. d.) Comparison of the average and standard deviation of Bray-Curtis distance metric for technical and biological replicates as compared to the average and standard deviation of all samples. This demonstrates that most of the variability in the dataset is not due to methodological errors. (TIF 1362 kb) [file 40168_2018_556_MOESM9_ESM.tif]
